# Authoritarian-Benevolent Leadership, Moral Disengagement, and Follower Unethical Pro-organizational Behavior: An Investigation of the Effects of Ambidextrous Leadership

**DOI:** 10.3389/fpsyg.2020.00590

**Published:** 2020-04-21

**Authors:** Kang-Hwa Shaw, Na Tang, Hung-Yi Liao

**Affiliations:** ^1^School of Management, Shandong University, Jinan, China; ^2^School of Management, Lanzhou University, Lanzhou, China; ^3^College of Philosophy, Law and Political Science, Shanghai Normal University, Shanghai, China

**Keywords:** ambidextrous leadership, authoritarian leadership, benevolent leadership, moral disinterment, unethical pro-organizational behavior

## Abstract

Drawing on the social cognitive theory of moral disengagement, this study examined the influence of the authoritarian-benevolent style of ambidextrous leadership on follower unethical pro-organizational behavior (UPB), mediated via moral disengagement. We tested the hypotheses using a sample of 175 participants at two time points. The results indicated that authoritarian-benevolent leadership affects moral disengagement. In addition, followers in congruent dyads with low authoritarian-benevolent leadership perceived higher levels of moral disengagement than those in congruent dyads with high authoritarian-benevolent leadership. Furthermore, high authoritarian-benevolent leadership had an indirect effect on follower UPB via moral disengagement. Theoretical and practical implications and future research directions are suggested.

## Introduction

Leaders may pay close attention to either ensuring that duties are performed or the well-being of personnel and need to choose between self-centric or follower-centric approaches to dealing with the different demands of followers in organizations. Previous studies showed that a single leadership style can no longer satisfy the diverse needs of organizations and their members. The effectiveness of ambidextrous leadership can drive organizations or leaders to manage multiple conflicts in organizations (Rosing et al., 2011). Ambidextrous leadership is a kind of behavior that manages tension or paradoxical situations and can judge, integrate, and coordinate two contradictory and complementary leadership styles according to the requirements of specific situations (Rosing et al., 2011; Luo et al., 2018). Moreover, it is regarded as an important driving force affecting follower outcomes. Current studies of ambidextrous leadership focus on normative, cognitive, and power perspectives. The normative perspective refers to believing that conventions are the foundation of an organization to operate, develop, and change in both exploration and exploitation strategies (Keller and Weibler, 2015). Thus, leaders of an organization must balance maintaining and breaking the system, and adapt to changes in the external environment through the complementarity of transformational leadership and transactional leadership (Schreuders and Legesse, 2012). The cognitive perspective emphasizes that leaders should select opening and closing leadership behaviors according to the requirements of different situations. While encouraging followers to break up routines, they need to establish rules to reduce possible risks (Rosing et al., 2011). The power perspective refers to accentuating loose and tight leadership models to affected work attitudes. Loose (participative) practices can increase follower flexibility and tight (directive) practices can improve execution efficiency. Consequently, leaders should complement and coordinate loose vs. tight leadership styles based on understanding of their differences (Sagie and Zaidman, 2002). Ambidextrous leadership involves maintaining a balance between the need to ensure that followers comply with organization requirements and ensuring that their needs are taken care of. To advance this line of research, we explore the effectiveness of authoritarian leadership and benevolent leadership on followers from the cognitive perspective.

We, therefore, consider that the leader should understand holistic thought that encompasses paradoxes (i.e., contradictions or yin-yang thought) in the organization. Indeed, a leader in the organization tends to contemplate management issues from the perspective of paradox integration (Chen, 2002); thus, the leader would be more likely to combine two opposite leadership styles. Leaders will both establish the authority to supervise their followers and show benevolence to them, thus presenting authoritarian-benevolent leadership (Cheng et al., 2004). The authoritarian-benevolent leadership style is strict while giving full attention to one’s followers. Followers will strive to achieve their goals to repay the support and trust of their leaders (Pellegrini et al., 2010). If an authoritarian leader also shows care for followers at the same time, that is, if the leader is also benevolent, it will reduce negative perceptions of authoritarian leadership by followers (Cheng et al., 2004). Therefore, followers may consider that the leader’s strictness is necessary to achieve organizational goals. Normally, in the running of organizations, followers will respond to different leadership styles. When leaders engage in benevolent leadership, their followers will show positive attitudes and behaviors, such as increased performance (Chen et al., 2014) and organizational citizenship behavior (Podsakoff et al., 2000). On the other hand, followers will have negative responses when a leader exerts authority, such as reduced loyalty (Lin et al., 2014). Therefore, authoritarian leadership and benevolent leadership are individually likely to elicit totally opposite attitudes and behaviors. Moreover, a leader’s behavior is often dual-natured and cannot be isolated in management practice (Zacher and Rosing, 2015). In prior research, authoritarian leadership combined with benevolence has shown a positive influence on follower performance (Cheng et al., 2004; Wang et al., 2016). An examination of the literature on ambidextrous leadership shows that previous research has focused on performance (Luu, 2017), innovation (Zacher et al., 2016; Berraies and El Abidine, 2019), and trust (Breevaart and Zacher, 2019), but there is a paucity of studies investigating the relationship between ambidextrous leadership and the external-role behavior of followers [e.g., unethical pro-organizational behavior (UPB)]. Conversely, prior studies have examined single leadership influence on follower UPB (e.g., ethical leadership “Miao et al., 2013; Kalshoven et al., 2016,” transformational leadership and transaction leadership, “Effelsberg et al., 2014; Graham et al., 2015”). However, thus far, no studies have focused on the influence of ambidextrous leadership on follower UPB, especially in relation to the ambidextrous leadership of authoritarian-benevolent leadership. UPB has both positive (pro-organizational) and negative (unethical) connotations. It is unknown what influence authoritarian-benevolent leadership has on UPB.

In recent years, instances of UPB have taken place in domestic and foreign enterprises, which has become a key issue of concern to all stakeholders (Trevino et al., 2014). UPB refers to followers’ unethical behavior that benefits their organization or colleagues (Umphress et al., 2010). Selfish unethical behavior and UPB can be distinguished by their intention. Selfish unethical behavior is when a follower acts out of self-interest (Kish-Gephart et al., 2010) or to harm others (Thau et al., 2007). On the other hand, followers engage in UPB for altruistic rather than self-interested motives, aiming to make the organization operate effectively (Umphress and Bingham, 2011). Ambidextrous leadership involves managing tension or paradoxical situations. Compared with a single leadership style, ambidextrous leadership involves more flexibility, situational dependence, contradiction balance, and inclusive thought (Rosing et al., 2011), which are more suitable for the paradoxical situation of follower UPB. It is important to explore the effects of ambidextrous leadership on UPB to further investigate the effectiveness of authoritarian-benevolent leadership.

Nonetheless, a few pieces of research have examined the interpersonal process, which is the top-down effect of authoritarian-benevolent leadership on followers’ organizational outcomes (Lin et al., 2014). Scholars have suggested that considering interpersonal processes may be valuable when examining the influence of leadership style on employee organizational outcomes (e.g., Huang et al., 2016). To better understand the impact of authoritarian-benevolent leadership in organizations, this study takes an interpersonal approach to consider how authoritarian-benevolent leadership influences followers’ UPB and its underlying mechanism. At present, an extensive body of research indicates that moral disengagement mediates the self-centered antecedents of unethical behaviors, such as empathy, moral identity, and envy (Detert et al., 2008; Duffy et al., 2012). In addition, Chen et al. (2016) claim that the moral disengagement can mediate the effects of prosocial antecedents, such as UPB. Although similar mechanisms (labeled *moral neutralization*) emerged in earlier theoretical models of Umphress and Bingham (2011), subsequent empirical research focused on distal antecedents, failing to take into account underlying psychological processes (Miao et al., 2013; Thau et al., 2015; Zhang Y. et al., 2018). Our study follows Chen et al.’s (2016) assertion that moral disengagement supports not only pro-self but also UPB. Specifically, based on the social cognitive theory of moral disengagement, our study investigates the impact of perceived leader authoritarianism and benevolence on followers’ UPB via moral disengagement, which refers to a set of interrelated cognitive mechanisms that can deactivate the moral self-regulation process to permit unethical behavior (Bandura, 1999). Previous research demonstrates the influence of moral disengagement on the organizational deviance on behalf of individuals (Zhang P. et al., 2018). This line of reasoning proposes that the moral disengagement of individuals can influence their own moral decisions and behaviors (Bandura, 1986). Specifically, individuals may redefine their unethical actions in order to minimize their own responsibility and the potentially harmful consequences of their unethical decision-making (Kish-Gephart et al., 2010). Thus, the aim of this current study is to deepen the application of the concept of moral disengagement to assess how follower UPB is affected by the leadership. Based on previous findings, we propose that follower moral disengagement may mediate the effect of authoritarian-benevolent leadership on follower UPB.

Overall, this study makes several contributions to the current literature. First, we add to the leadership literature by examining the authoritarian-benevolent style of ambidextrous leadership. As previous research has focused on transformational leadership and transactional leadership to test ambidextrous leadership (Schreuders and Legesse, 2012), we examined authoritarian-benevolent leadership from the cognitive perspective as well as its mixed effects within an organization. Thus, investigating authoritarian-benevolent leadership within organizations could have theoretical and practical implications. Second, this study contributes to a deeper understanding of the relationship between authoritarian-benevolent leadership and follower UPB by adopting an interpersonal approach. Previous studies on single leadership styles examined the influence of either authoritarian leadership or benevolent leadership on followers’ outcomes (e.g., performance, Cheng et al., 2004; organizational citizenship behavior, Tang and Naumann, 2015). Our study investigated the influences of an authoritarian-benevolent style of ambidextrous leadership on follower UPB, which helps to provide a more comprehensive understanding of the effects of leaders’ “authoritarianism-benevolence” and followers’ “good-bad” behavior (i.e., behavior that is simultaneously unethical yet also pro-organizational) within organizations. Third, we contribute to the literature on authoritarian-benevolent leadership by revealing the underlying social cognitive mechanism. This study adopts a social cognitive perspective and reveals followers’ moral disengagement as the psychological mechanism underlying the relationship between authoritarian-benevolent leadership and follower UPB. Therefore, our study provides a new theoretical perspective for the research field of leader-follower dynamics.

## Theoretical Background and Hypotheses

### Authoritarian-Benevolent Leadership

Authoritarian-benevolent leadership is an ambidextrous leadership style in organizations. In the workplace, authoritarian leadership concentrates on followers’ performance, and the leader asks his/her followers to obey orders; benevolent leadership shows holistic concern for followers’ well-being and cares for them within the workplace (Farh and Cheng, 2000; Farh et al., 2008). Therefrom, from the perspective of ambidextrous leadership, we define authoritarian-benevolent leadership (the coexistence of authoritarian leadership and benevolent leadership) as when leaders show two complementary leadership behaviors, authority and benevolence, and can coordinate the use of these two leadership behaviors according to their situations.

Authoritarian leadership can be conceptualized as leaders’ behaviors that exert control over followers, exercise absolute authority, and demand unconditional obedience (Cheng et al., 2004). When leaders implement an authoritarian approach to their followers or followers are demanded to comply with their leaders’ requests, followers might have negative feelings toward leaders (Farh et al., 2006). Previous studies indicated that authoritarian leadership has negative effects on followers’ attitudes and behaviors, such as voice behavior (Li and Sun, 2015) and performance (Wu M. et al., 2012; Wu T.Y. et al., 2012; Chan et al., 2013); however, some studies claim that there is a positive relationship between authoritarian leadership and follower performance (Wang and Guan, 2018). The explanation of the mixed findings regarding authoritarian leadership and follower outcomes may involve the underlying psychological processes. The extant mechanisms have not been clearly explored enough to provide a full picture of the actual influence of authoritarian leadership (Cheng et al., 2004). Thus, when an authoritarian leader shows leniency to his/her followers, they will have different feelings in the psychological process. This may explain why authoritarian leaders have varying influences on their followers.

Benevolent leadership refers to a behavior that involves long-term concerns for followers’ performance in the workplace and personal well-being in life (Cheng et al., 2000). It is an effective leadership style that represents an obligation and positive action to one’s follower in the organization that encourages them to reciprocate and comply with leader requests (Chan et al., 2013). That is, benevolent leadership has a positive influence on follower attitude and behavior, such as fostering loyalty and hard work (Shin et al., 2012), trust (Wasti et al., 2011), improved performance (Chan and Mak, 2012; Chan et al., 2013), and innovative behavior (Gumusluoglu et al., 2017). In addition, benevolent leadership, including ethical sensitivity, refers to leaders’ consideration that what is right or wrong and the process of moral reflection at work (Karakas and Sarigollu, 2012) could influence the moral behavior of followers. Moreover, Cheng and Wang (2015) claim that benevolent leadership influences on team identification via an ethical climate. Recent studies have investigated positive relationships of benevolent leadership with deviant behavior, such as pro-social rule-breaking (Li et al., 2015), and negative effects on team performance (Li et al., 2018). Therefore, in view of the dark side of benevolent leadership, it is important for leaders to conscientiously monitor and standardize their followers’ behavior. The complex findings on authoritarian leadership and benevolent leadership have prompted calls to further investigate (1) the coexistence of authoritarian leadership and benevolent leadership, which may explain the effectiveness of such leadership; and (2) psychological mechanisms underlying authoritarian-benevolent leadership’s effect on follower outcomes.

### Authoritarian-Benevolent Leadership and Moral Disengagement

To advance this line of research, we take a follower-centered perspective to explore the psychological process that links authoritarian-benevolent leadership to follower behavior. From this perspective, we can better understand how leaders shape follower behavior through the self-construction and experiences of the follower. Social cognitive theory proposes that personal cognition is determined by the interaction between situational and personal factors (Bandura, 1986). According to the social cognitive theory of moral disengagement, individuals exert cognitive control over their feelings, thoughts, and behaviors based on their own internal and external moral standards about how one should behave (Bandura et al., 1996). Moreover, moral disengagement is not an invariable individual cognitive mechanism; it can be shaped by situational factors (Fida et al., 2015; Petitta et al., 2017), and moral self-regulation processes can be deactivated by a series of cognitive mechanisms elicited by specific environmental demands to behave in ways that conflict with ethical values (Bandura, 1991; Moore, 2008). It specifies eight mechanisms of moral disengagement that are grouped into three sets (Bandura, 1999) that facilitate the justification of deviant or unethical behaviors (Beu and Buckley, 2004; Zhang P. et al., 2018).

The first set of moral disengagement mechanisms concerns the cognitive construal of conduct, which includes moral justification, euphemistic labeling, and advantageous comparison (Bandura, 1999; Bandura et al., 2001), and through these processes, unethical and immoral behavior will be described as less harmful. The second set of disengagement mechanisms comprises displacement of responsibility, diffusion of responsibility, and ignoring/misconstruing of consequences of the action to hold individuals aloof from the harmful outcomes (Bandura et al., 2001). The third set is made up of dehumanizing the victim or attributing blame to the victim (Bandura, 1986), and focuses on the recipient of the immoral or unethical behaviors. When individuals dehumanize the victim, they can lessen their identification with the victim of their unethical behavior and feel less immoral.

Drawing on the social cognitive theory of moral disengagement, we propose that perceived authoritarian-benevolent leadership will cause followers’ moral disengagement for three reasons. First, previous studies have dealt with the positive effects of authoritarian leadership and benevolent leadership on follower deviant behaviors such as unethical decision making (Hing and Ramon, 2007), turnover intention (Wang et al., 2018), and pro-social rule breaking (Li et al., 2015). Leaders are conceived as role models for their followers and always as delegates of their organizations. Under authoritarian-benevolent leadership, followers would perceive that these harmful misbehaviors are acceptable to organizations and individuals, so they will reinterpret abnormal behaviors as less harmful ones. Moreover, compared with the results of leadership behaviors, followers will deem their own unethical behavior, ranging from theft to cheating, as more insignificant and less likely to have serious consequences. Second, in organizations, a follower’s aberrant behaviors seem to be negligible, implicit, and unobtrusive, and so these behaviors cannot be easily observed and detected. Thus, when an individual’s role in harmful effects is ambiguous or can be attributed to others, more than likely, he/she will morally disengage. In addition, according to social identity theory (Ashforth and Mael, 1989), authoritarian leadership will influence follower organizational citizen behavior via collectivism (Ning et al., 2012); benevolent leadership would enhance follower recognition (Cheng et al., 2004), leading to negative behaviors such as corruption (Ashforth and Anand, 2003). Consequently, because followers construe their behavior in terms of collective action, for which no individual will be blamed, they are more likely to feel a displacement of responsibility (Bandura, 1991).

Last, to a certain extent, moral disengagement depends on how the actor views the recipients as the target of the behaviors. Authoritarian leadership has been found to be powerful and controlling (Farh and Cheng, 2000), but when followers perceive work pressure and exploitation by leaders, they will experience anger and give unfavorable returns (Harvey et al., 2016); although benevolent leadership has also been demonstrated to involve holistic concern for followers (Farh and Cheng, 2000), to obtain more care and solicitude from benevolent leaders in the follow-up process, followers will work harder and be loyal to their leaders (Shin et al., 2012), regardless of the interests of other stakeholders. Therefore, it can be reasonably assumed that when followers have interactions with authoritarian-benevolent leadership, they would increasingly resist authority and show loyalty, and be more likely to dehumanize their authoritarian-benevolent supervisors or attribute blame to their leaders. To sum up, we predict the following:

Hypothesis 1: The more aligned the levels of authoritarian leadership and benevolent leadership are (i.e., leadership is equally authoritarian and benevolent), the greater the moral disengagement.

As indicated earlier, ambidextrous leadership integrates two different complementary leadership behaviors: authoritarian leadership and benevolent leadership (Rosing et al., 2011). We, therefore, follow a “both-and” method to determine dynamic balance and coordinated development of contradictory behaviors (Gebert et al., 2010). Moreover, when authoritarian leadership is assisted by caring and solicitous behaviors, followers will feel gratitude toward their leaders, which reduces the possible harmful effects of authoritarian leadership (Cheng et al., 2002; Chou et al., 2010). Additionally, when benevolent leadership shows authority, followers will be understanding of this severe behavior and, in good faith, consider it to be a high standard and demanding task, not a personal sentiment toward followers. Authoritarian leaders and benevolent leaders often produce diametrically opposite leadership effectiveness (authoritative behaviors induce negative work psychology and behavior in followers, and benevolent leaders encourage followers to actively engage in work); however, these qualities also exist in the management behavior of leaders (Pellegrini and Scandura, 2008). Thus, this study focuses on leadership that is equally authoritarian and benevolent.

Leaders may show different degrees of authority and benevolence, which lead to changes in followers’ psychological perceptions involving moral disengagement. Specifically, when authoritarian-benevolent leadership is at a high level, on the one hand, the leader seizes all the power to enable followers to actually execute the work instructions (Cheng et al., 2004). Also, they have power over followers, which further induces followers’ compliance and obedience. Additionally, authoritarian leaders can enhance the affective trust (Tian and Sanchez, 2017) to create interdependence between followers and their leaders; on the other hand, the leader also takes care of his/her followers inside and outside the workplace (Farh and Cheng, 2000). Moreover, benevolent leaders transmit their role expectations to their followers and tend to use friendly and compassionate actions to inspire followers’ sense of responsibility and loyalty to their expected roles. In addition, benevolent leaders can instill trust in their followers (Wasti et al., 2011). As discussed above, when followers perceive leaders with authoritarianism as benevolent, they are appreciative of and understand the style of authoritarian leadership. In this condition, followers will strictly abide by the rules and repay the favors of their leader, and display fewer deleterious behaviors.

When authoritarian-benevolent leadership is at a low level, followers observe and endure an unfriendly leadership style, which is low in benevolence, and they sense that they can do whatever they want. That is, leaders do not pay attention to their followers inside and outside the workplace. The low level of leader authority prompts followers to slacken their vigilance and become negligent in the workplace (Ren et al., 2003). Thus, if leaders provide less care and guidance at the same time, it elicits followers’ inertia. Specifically, followers recognize their leaders’ decreased care and counseling simultaneously, so that in this situation, followers “get away” with loafing on the job, and even consider their own interests at the expense of the organizational rules, which leads to self-interest behavior. In this type of organizational atmosphere, which lacks supervision and warmth, the followers are likely to espouse a self-interested perspective, rather than feeling any personal obligations toward the organization. In this condition, followers would consider their behavior as being of no concern and unregulated, and then they reinterpret and perceive their own self-interest unethical behaviors as more acceptable behaviors. Hence, we propose the following:

Hypothesis 2: Moral disengagement is higher when leadership is low in both authoritarianism and benevolence (i.e., low authoritarian-benevolent leadership) rather than when benevolent leadership is aligned with a high level of authoritarian leadership (high authoritarian-benevolent leadership).

### Moral Disengagement as a Mediator of the Effect of Authoritarian-Benevolent Leadership on Follower UPB

According to the social cognitive theory of moral disengagement, moral disengagement deactivates normative action and moral self-regulation processes (Bandura, 1991), so that people violate their own moral standards without self-condemnation for performing a series of transgressive and unethical behaviors, such as UPB (Detert et al., 2008; Barsky, 2011; Moore et al., 2012; Chen et al., 2016). Moreover, moral disengagement is theorized as a pre-transgression justification (Ribeaud and Eisner, 2010). Thus, we propose that followers high in moral disengagement may engage in follower UPB. We deem that when followers face a situation involving moral dilemmas related to the organization’s interests, ambidextrous (authoritarian-benevolent) leadership can lead to follower UPBs by activating moral disengagement, thereby eliminating self-regulation and self-condemnation of harmful conduct, and encouraging self-approval of unethical behavior. This might be the followers’ explicit justification of unethical behavior as necessary to take orders from their leader to protect organizational interests or may also cover the unethicality of lying by euphemistic language to gain credit from their leaders. Therefore, when followers perceive authoritarian-benevolent leadership, the leader’s authority and benevolence enhance followers’ UPB via moral disengagement. Hence, we propose the following hypothesis:

Hypothesis 3: Moral disengagement mediates the relationship between authoritarian-benevolent leadership and follower UPB.

## Materials and Methods

### Participants and Procedures

Data were collected from multiple companies that provided IT services, located in Shandong province, China. We followed the suggestion of Podsakoff and Organ (1986) to ask each respondent to anonymously return the survey directly to us after completing it. We collected the data at two points in time. In the first survey, we collected 213 questionnaires to measure authoritarian leadership, benevolent leadership, and moral disengagement. After 4 weeks, we measured follower UPB and finally received 175 surveys (82.16% response rate). Among the respondents, 53.14% were female, the average age was 27.75 years old (SD = 7.12), the average tenure was 4.02 years (SD = 7.23), and 64% had college degrees (SD = 0.81).

### Measures

Most measurement scales were originally written in English; thus, we translated them into Mandarin Chinese using Brislin’s (1986) “back translation” procedures. All ratings were measured on a scale from 1 (“strongly disagree”) to 6 (“strongly agree”).

#### Authoritarian-Benevolent Leadership

We adopted Cheng et al.’s (2002) 20-item scale that includes a 9-item authoritarian leadership scale and an 11-item benevolent leadership scale to measure the leader’s authority and benevolence, respectively. Sample items include “My supervisor will help me when I am in an emergency,” and “My supervisor asks us to obey his/her instructions completely.” The Cronbach α was 0.85 for authoritarian leadership and 0.91 for benevolent leadership.

#### Moral Disengagement

Moral disengagement was measured with Moore et al.’s (2012) eight-item moral disengagement scale, which is useful for the assessment of adults in the context of the workplace. Sample items include “It is okay to spread rumors to defend those you care about,” and “People should not be held accountable for doing questionable things when they were just doing what an authority figure told them to do.” The Cronbach α was 0.91 for this scale in our study.

#### Unethical Pro-organizational Behavior

We used Umphress et al.’s (2010) six-item UPB scale. Sample items include “If it would help my organization, I would misrepresent the truth to make my organization look good,” and “If it would benefit my organization, I would withhold negative information about my organization.” The Cronbach α was 0.88 for this scale in our study.

#### Control Variables

As prior research has shown that demographic characteristics may influence the extent to which individuals engage in unethical behavior (Kish-Gephart et al., 2010; Umphress et al., 2010), we controlled for the effects of demographic characteristics (i.e., gender, age, education, and tenure). All scales included in the surveys can be found in the Supplementary Material.

### Analytical Approach

We used SPSS 22.0 to analyze the correlations and regressions and conducted a set of confirmatory factor analyses (CFAs) using AMOS 18.0. To test hypotheses 1 and 2, we used the polynomial regression and response surface methodology (Edwards and Parry, 1993; Zhang et al., 2012). Compared with moderating regression and difference value analysis, this methodology can provide more accurate results. The formula is shown below:

(1) $$\begin{array}{c} Z=b_{0}+b_{1}(X)+b_{2}(Y)+b_{3}(X^{2})+b_{4}(X\times Y)+\\ \;b_{5}(Y^{2})+e \end{array}$$

In the formula, *Z* represents moral disengagement, *X* is benevolent leadership, and *Y* is authoritarian leadership. Hypotheses 1 and 2 suggest that authoritarian-benevolent leadership influences follower moral disengagement, so we used the estimated coefficients as well as the slopes and curvatures along the (in)congruence line for the polynomial regressions in predicting moral disengagement. To test the indirect effects of authoritarian-benevolent leadership on follower UPB via moral disengagement (hypothesis 3), we chose the block variable approach (Edwards and Cable, 2009). Specifically, based on the polynomial regressions predicting moral disengagement, we calculated a single coefficient (e.g., the block variable) representing the effects of leader authority and benevolence on moral disengagement. We next use bootstrapping to examine the 95% confidence interval (CI) of the mediation effect of moral disengagement between authoritarian-benevolent leadership and UPB.

## Results

### Measurement Model Analysis

Before testing our hypotheses, we conducted CFAs to examine the discriminant validities (Anderson and Gerbing, 1988). Results showed that a four-factor model (χ^2^/df = 2.18, RMSEA = 0.08, NFI = 0.87, NNFI = 0.92, CFI = 0.93, IFI = 0.93) had a superior model fit over a one-factor model (χ^2^/df = 8.09, RMSEA = 0.20, NFI = 0.70, NNFI = 0.73, CFI = 0.75, IFI = 0.75), as shown in Table 1.

**TABLE 1:** Comparison of factor structures.

Model	χ ^2^	df	Δχ ^2^	RMSEA	NFI	NNFI	CFI	IFI
Model 1	1136.28	521	–	0.08	0.87	0.92	0.93	0.93
Model 2	2016.82	522	880.54**	0.13	0.82	0.86	0.87	0.87
Model 3	3724.11	524	2587.83**	0.19	0.73	0.76	0.78	0.78
Model 4	4262.09	527	3125.81**	0.20	0.70	0.73	0.75	0.75

N = 175. Model 1 is the hypothesized four-factor model; model 2 is a three-factor combining authoritarian leadership and benevolent leadership; model 3 except UPB, combining in a one-factor model; model 4 is a single-factor model combining all variables. **p < 0.01.

We conducted Harman’s one-factor test by using exploratory factor analysis for all the independent variables (except demographics) to check common method bias (Podsakoff et al., 2003). The first factor only accounted for 24.65% of the total variance. No single major factor emerged to explain the majority of the variance involved in the model, and the results showed that no substantial common method bias existed in the data. As Harman’s one-factor test and the CFA both showed, the variables were distinct; thus, common method bias was not a problem in this study.

### Descriptive Statistics

We present the means, standard deviations, and correlations among all the variables in Table 2. The results show that authoritarian leadership was positively related to UPB (*r* = 0.28, *p* < 0.01); benevolent leadership was positively related to UPB (*r* = 0.17, *p* < 0.05); authoritarian-benevolent leadership was positively related to UPB (*r* = 0.20, *p* < 0.01); authoritarian-benevolent leadership was positively related to moral disengagement (*r* = 0.26, *p* < 0.01); moral disengagement was positively related to UPB (*r* = 0.68, *p* < 0.001).

**TABLE 2:** Means, standard deviations, and correlations.

Variable	Means	SD	1	2	3	4	5	6	7	8
1. Gender	1.53	0.50								
2. Age	27.75	7.12	−0.13							
3. Education	2.86	0.81	−0.07	−0.26**						
4. Tenure	4.02	7.23	−0.07	0.40**	−0.16*					
5. Authoritarian leadership	3.62	0.88	−0.21**	0.08	−0.14	0.12				
6. Benevolent leadership	3.86	0.96	−0.06	−0.16*	−0.02	−0.17*	−0.01			
7. Authoritarian-benevolent leadership	13.96	5.18	−0.21**	−0.08	−0.12	−0.07	0.68**	0.71**		
8. Moral disengagement	2.51	1.05	−0.23**	−0.10	0.15*	−0.07	0.29**	0.09	0.30**	
9. Unethical pro-organizational behavior	2.89	1.10	−0.29**	−0.18*	0.27**	−0.15	0.28**	0.17*	0.34**	0.69**

N = 175; *p < 0.05, **p < 0.01; authoritarian-benevolent leadership is the interaction of authoritarian leadership and benevolent leadership.

### Hypothesis Testing

**FIGURE 1:**
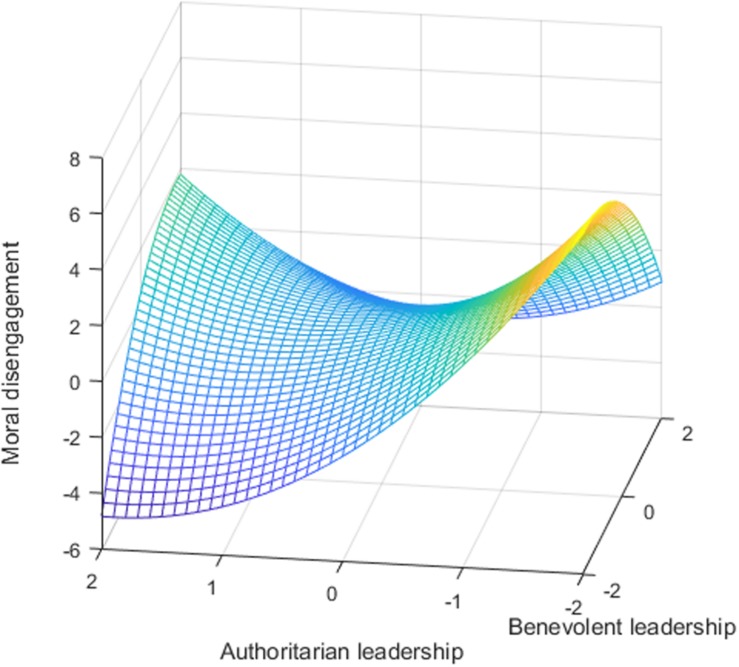
The effect of authoritarian-benevolent leadership on moral disengagement.

To test hypothesis 1, Table 3 shows the results of polynomial regression. Based on the results of model 3 in Table 3, our study tested the response surface methodology, as shown in Table 4. As shown in Table 4, the surface along the (in)congruence line was curved (curvature = 1.1, *p* < 0.05). Figure 1 also indicates an inverted U-shaped curve along the incongruence line. The concave curvature along the *X* = −*Y* line shows that moral disengagement was higher when authoritarian leadership was aligned with benevolent leadership, and any deviation from the congruence line increased moral disengagement; hypothesis 1 was supported. Second, to test hypothesis 2 (moral disengagement is higher when authoritarian-benevolent leadership is low than when authoritarian-benevolent leadership is high), the results show that the slope of consistency (*X* = *Y*) was significant (slope = −1.06, *p* < 0.01), illustrating that when authoritarian-benevolent leadership was low, followers had more moral disengagement. Thus, hypothesis 2 was verified.

**TABLE 3:** Polynomial regression.

	Moral Disengagement		
	M1	M2	M3
Constant	0.69	0.28	0.05
Gender	−0.48*	−0.34*	−0.26
Age	−0.01	−0.01	−0.01
Education	0.13	0.19*	0.19*
Tenure	−0.01	−0.01	−0.00
Independent variable			
Benevolent leadership (*b*_1_)		0.06	−0.07
Authoritarian leadership (*b*_2_)		0.28**	−0.99*
Benevolent leadership^2^ (*b*_3_)			−0.49
Authoritarian-benevolent leadership (*b*_4_)			0.86*
Authoritarian leadership^2^ (*b*_5_)			0.73
*R*^2^	0.09	0.16	0.21
Adj. *R*^2^	0.06**	0.13**	0.16*
*F*	3.77	5.30	4.78

*p < 0.05, **p < 0.01.

**TABLE 4:** Test of response surface coefficient.

Estimate	Moral disengagement
Consistency *X* = *Y*	
Slope (*b*_1_ + *b*_2_)	−1.06*
Curvature (*b*_3_ + *b*_4_ + *b*_5_)	1.1*
Inconsistency *X* = -*Y*	
Slope (*b*_1_ – *b*_2_)	0.92*
Curvature (*b*_3_ – *b*_4_ + *b*_5_)	−0.62*

N = 175. *p < 0.05.

Finally, to test the indirect effect of authoritarian-benevolent leadership with UPB via moral disengagement (hypothesis 3), we referred to the suggestion of Edwards and Cable (2009) to use the block variable approach. Specifically, this was used to estimate the path from the authoritarian-benevolent leadership polynomial terms to moral disengagement. More importantly, the use of block variables does not change the evaluation coefficients and total interpretation rates of other variables in the equation (Zhang et al., 2011). We created a block variable by multiplying the estimated polynomial regression coefficients (from the moral disengagement regression described above) with the raw data to obtain a weighted linear composite. We then used bootstrapping to test the indirect effect of authoritarian-benevolent leadership on UPB (Zhao et al., 2010). The results of the analyses examining the indirect effect of authoritarian-benevolent leadership on UPB via moral disengagement at conditional values indicate that the indirect effect of authoritarian-benevolent leadership was 0.06, the 95% bias-corrected bootstrap CI excluded 0 (0.08, 0.30), high authoritarian-benevolent leadership was 0.08 (CI: 0.00, 0.28), and low authoritarian-benevolent leadership was 0.05 (CI: −0.02, 0.15), showing that the mediation effect at high authoritarian-benevolent leadership was significant in Table 5. Thus, hypothesis 3 was supported.

**TABLE 5:** Results from test of indirect effect of authoritarian-benevolent leadership on UPB.

Variables	Authoritarian-benevolent leadership to moral disengagement	Moral disengagement to UPB	Indirect effect of authoritarian-benevolent leadership to UPB 95% CI
Authoritarian-benevolent leadership (block variable)	0.32**	0.58***	0.06 [0.08, 0.30]
High authoritarian-benevolent leadership (block variable)	0.21**	0.61***	0.08 [0.00, 0.28]
Low authoritarian-benevolent leadership (block variable)	0.13*	0.61***	0.05 [−0.02, 0.15]

Note: Significance of bootstrapped indirect effect was determined by examining the bias-corrected 95% CI for the indirect effect using 5,000 bootstrap samples. *p < 0.05, **p < 0.01, ***p < 0.001.

## Discussion

### Theoretical Implications

In the present study, based on the social cognitive theory of moral disengagement (Bandura, 1999), we proposed and tested the effect of authoritarian-benevolent leadership on follower UPB via the indirect effect of moral disengagement. Our study makes three contributions.

First, our research contributes to the leadership literature field. Current research in this field has predominantly focused on linking outcomes of leadership with positive paradigms (Cheng et al., 2004; Chan et al., 2013), but the conclusions of the studies were mixed. For example, some research claimed that authoritarian-benevolent leadership has a positive influence on follower performance (Wang et al., 2016), while another study had the opposite result (Li et al., 2014). These studies tested the interaction of authoritarian leadership and benevolent leadership. However, this study investigated the influence of the authoritarian-benevolent style of ambidextrous leadership on follower moral disengagement. Our findings illustrate that authoritarian-benevolent leadership is positively related to followers’ moral disengagement (hypothesis 1). This result is consistent with previous research that indicates that authoritarian leadership is related to moral disengagement (Liu et al., 2012), and the argument that followers may consider authoritarian-benevolent leadership is regulated and shows greater tolerance for this style of leadership. Therefore, the results of our study extend the leadership literature concerning the actual effect of authoritarian-benevolent leadership. Alternatively, we attempted to reveal alternate possibilities and expand previous research on ambidextrous leadership (Rosing et al., 2011; Schreuders and Legesse, 2012; Keller and Weibler, 2015). Thus, research on authoritarian-benevolent leadership helps in discovering untapped fields in regard to ambidextrous leadership that consider the negative effects of such leadership, thus offering a deeper understanding of the topic.

Second, we contributed to the study of leadership by adopting a follower-centered perspective and a psychological perspective to discuss the link between authoritarian-benevolent leadership and moral disengagement. Previous research has investigated the mechanism and effects of authoritarian leadership or benevolent leadership on followers’ destructive behaviors (cf. Li et al., 2015; Harvey et al., 2016). Adopting this line did help provide comprehensive insight into authoritarian leadership and benevolent leadership in organizations. However, to solely focus on both leadership styles individually would inevitably limit our study scope. By considering ambidextrous leadership, our research showed how authoritarian-benevolent leadership emerges as leaders can induce moral disengagement.

Third, we uncovered a unique and important psychological mechanism to explain the effects of authoritarian-benevolent leadership on followers’ UPB by adopting a social cognitive perspective. Although research has utilized social identity theory to explain that leaders’ UPB or leadership style might cause another party’s UPB (Umphress et al., 2010; Miao et al., 2013; Zhang Y. et al., 2018), social identity theory does not explain the mechanisms between ambidextrous leadership and UPB. In addition, the leadership style is multivariate, and social cognitive theory advocates that the psychological mechanism that influences individual behavior through cognition may easily happen and more accurately explain the actual situation. Drawing upon social cognitive theory, our study illuminates that authoritarian-benevolent leadership could trigger followers’ deactivation of ethical self-regulation. Therefore, we contributed to the study of authoritarian-benevolent leadership by adopting a social cognitive perspective to examine the dynamics between this leadership style and follower’s behavior. Moreover, by investigating the relationship between authoritarian-benevolent leadership and follower UPB, we shed light on not only ambidextrous leadership but also the psychological mechanisms through which leadership styles interfere with followers’ behaviors.

### Practical Implications

This research provides several valuable managerial implications for organizations. First, leaders must give thought to analyzing and discussing ethical problems in organizations. Thus, authoritarian-benevolent leadership is one style of ambidextrous leadership. Leaders can provide effective ambidextrous leadership in different situations to integrate problems and manage the organization well. Second, leaders should not simply show benevolent leadership or authoritarian leadership but must attach great importance to and cultivate authoritarian-benevolent leadership. In particular, authoritarian-benevolent leadership influences follower performance (Lin et al., 2014), as leaders have the authority to request and support followers. Thus, in the process of requiring followers to work hard, they need to pay attention to their own communication, show correct conduct, care about followers, and encourage them to work actively. However, potential unethical intentions of followers will be improved. Third, leaders should pay attention to reducing the UPB of followers. Prior work on unethical pro-organization behaviors in organizations shows that follower UPB appears to be subtle and may be done for apparently good reasons (Umphress and Bingham, 2011). Therefore, UPB takes time to discover and is not easy to regulate. Authoritarian-benevolent leadership needs to combine long-term goals with the interests of followers to abate follower UPB.

### Limitations and Future Directions

Our research has several limitations and indicates several suggestions for future research. First, the data were collected from a single source who may have answered the sensitive self-report in a socially desirable way (e.g., UPB measure), which could introduce common method variance, despite the fact that we collected the data at two different time points. Therefore, future research could collect variables from different sources or conduct a longitudinal study and also follow the methodology of Chen et al. (2016) and Umphress et al. (2010) to control the effect of behaviors linked with social desirability. Second, while we tested the indirect effect of authoritarian-benevolent leadership on follower UPB via moral disengagement, we did not investigate all eight mechanisms of moral disengagement. Thus, future studies could examine separately those eight mechanisms of moral disengagement (Bandura, 1999) and expand the range of potential mediators, such as learning goal orientation (Wang and Guan, 2018). Third, in this study, we did not examine the boundary conditions on the effectiveness of authoritarian-benevolent leadership. Leaders need to respond to the variability of the workplace, and even the authoritarian-benevolent style of ambidextrous leadership can be effective in some situations. Therefore, future research must examine the boundary conditions to test the effectiveness of authoritarian-benevolent leadership, such as moral identity and leader identification (Zhang Y. et al., 2018).

## Data Availability Statement

The datasets generated for this study are available on request to the corresponding author.

## Ethics Statement

An ethics approval was not required per the author’s institutions’ guidelines and national regulations. For the participants, we sent online questionnaires, which directly link to individual members. All subjects gave written informed consent in accordance with the Declaration of Helsinki. The cover page of the questionnaire explained the study objectives, the voluntary nature of the survey, and an assurance of confidentiality to participants. We were exempt from further ethics board approval since this research did not involve human clinical trials or animal experiments.

## Author Contributions

K-HS was responsible for idea generation, manuscript writing for the theoretical part, and data collection. NT has been involved in idea generation and data analysis. H-YL was responsible for the initial method part writing. All authors reviewed and approved the manuscript for publication.

## Conflict of Interest

The authors declare that the research was conducted in the absence of any commercial or financial relationships that could be construed as a potential conflict of interest.

## Supplementary Material

TABLE S1 Authoritarian-benevolent leadership, moral disengagement, and follower unethical pro-organizational behavior: an investigation of the effects of ambidextrous leadership.
